# Preparation of Silk Sericin/Lignin Blend Beads for the Removal of Hexavalent Chromium Ions

**DOI:** 10.3390/ijms17091466

**Published:** 2016-09-02

**Authors:** Hyo Won Kwak, Munju Shin, Haesung Yun, Ki Hoon Lee

**Affiliations:** 1 Research Institute of Agriculture and Life Sciences, Seoul National University, Seoul 151-921, Korea; bk0502@snu.ac.kr; 2 Department of Biosystems & Biomaterials Science and Engineering, Seoul National University, Seoul 151-921, Korea; smj0626@snu.ac.kr (M.S.); finale17@snu.ac.kr (H.Y.); 3 Center for Food and Bioconvergence, Seoul National University, Seoul 151-921, Korea

**Keywords:** lignin, silk sericin, beads, adsorption, hexavalent chromium

## Abstract

In the present study, novel adsorbents having high adsorption capability and reusability were prepared using agricultural by-products: silk sericin and lignin. Silk sericin and lignin blend beads were successfully prepared using simple coagulation methods for the removal of hexavalent chromium (Cr(VI)) from aqueous solution. A 1 M lithium chloride (LiCl)/dimethyl sulfoxide (DMSO) solvent system successfully dissolved both sericin and lignin and had sufficient viscosity for bead preparation. Compared to the conventional sericin bead adsorbent, sericin/lignin blend beads showed higher Cr(VI) adsorption capacity. The amount of lignin added to the adsorbent greatly affected the adsorption capacity of the beads, and a 50:50 sericin/lignin blend ratio was optimal. Adsorption behavior followed the Freundlich isotherm, which means the adsorption of Cr(VI) occurred on the heterogeneous surface. Cr(VI) adsorption capability increased with temperature because of thermodynamic-kinetic effects. In addition, over 90% of Cr(VI) ions were recovered from the Cr(VI) adsorbed sericin/lignin beads in a 1 M NaOH solution. The adsorption-desorption recycling process was stable for more than seven cycles, and the recycling efficiency was 82%. It is expected that the sericin/lignin beads could be successfully applied in wastewater remediation especially for hazardous Cr(VI) ions in industrial wastewater.

## 1. Introduction

Several industrial processes require a variety of heavy metals, and the excessive discharge of heavy metals has been a major environmental problem. Most of the heavy metals are easily soluble in water, and they can be quickly accumulated in living organisms. The amount of heavy metals accumulated in the human body tends to increase gradually through the food chain. A high concentration of heavy metals is well known to adversely affect the human body. Among the various heavy metals, Cu, Cd, Hg, Zn and Cr are the typical hazardous metals that are produced by chemical-intensive industries [1].

Chromium is one of the most notorious heavy metals released by basic industries, such as the metallurgical, refractory and chemical industries. In the chemical industry, chromium is primarily used in leather tanning, electroplating, dyes and pigments and wood treatment. Small amounts of chromium are also widely used in catalysts, corrosion inhibitors, photography and manufacturing industries. Chromium has various oxidation states from −2 to +6 [2]. In aqueous solutions, the most commonly-observed oxidation states are +3 (trivalent) and +6 (hexavalent). The most prominent toxic oxidation state is +6 (Cr(VI)). Generally, Cr(VI) is considered 1000-times more toxic than Cr(III). Cr(VI) has been classified as carcinogenic to humans by the International Agency for Research on Cancer (IARC). Because of its carcinogenicity, the World Health Organization (WHO) recommended a maximum allowable concentration of 0.05 mg/L for Cr(VI) in drinking water, based on the health concerns [3]. There have been various Cr(VI) remediation techniques, including chemical precipitation, ion exchange, electro or chemical coagulation and the adsorption process. Among these, the adsorption technique is highly economical, because it can be use various inexpensive biomass-derived polymers as the adsorbent. Furthermore, the adsorption process could be reusable if the adsorption and desorption process is reversible. This reusability of the adsorbent could prevent the secondary pollution and facilitate the recovery of metal ions from the process. For practical development of this adsorption process, finding the appropriate adsorbent sources, which have a higher adsorption efficiency and recyclability, is important.

Silk is a protein-based natural polymer spun by a variety of species, including silkworms and spiders [4,5,6]. Silk fiber secreted by silkworms consists of two major proteins, namely fibroin and sericin. When a silkworm spins cocoon silk fibers, a glue-like sticky layer of sericin surrounds two filaments of fibroin for adhesive and protective purposes [7,8]. Sericin constitutes approximately 20%–30% (*w*/*w*) of the total cocoon silk fibers. In most silk industries, including the fiber industry, and biomedical applications, the sericin is removed to improve luster and biocompatibility. Sericin can be removed via a process called “degumming”, which uses an alkaline solution, high temperature and high pressure. The global silk production statistics show that the annual cocoon production in the 21st century is more than 170,000 metric tons [9]. In other words, close to 68,000 tons of sericin are discarded through the degumming wastewater solutions. Recently, sericin has been reported to have a variety of biofunctions, and currently, there are many efforts to utilize sericin in the polymeric and biomedical material fields [10,11,12,13,14].

Finding a good solvent for the fabrication of a natural polymer is very important for its applications. There are many solvent systems for the fabrication of sericin into various forms, including films, nanofibers, hydrogels and macro- to micro-particles [15,16,17,18]. In the cases of films and hydrogels, sericin/water systems have been widely used owing to the water solubility of sericin. However, molecular aggregation and fast gelation occur easily in sericin/water solutions; this makes the fabrication process difficult to control because of fast variations in viscosity. To overcome this solution instability of sericin/water, various alternative solvents to the conventional aqueous systems have been considered, such as formic acid and trifluoroacetic acid [19,20,21,22]. Um et al. prepared sericin films using a formic acid solution and found that formic acid retarded the gelation of sericin compared to aqueous sericin/water solutions [23]. Another alternative solvent system for the fabrication of sericin is 1 M lithium salt in dimethyl sulfoxide (DMSO). Oh et al. used a LiCl/DMSO solvent system to prepare sericin beads and found that sericin can be dissolved in this solvent system with approximately 30% LiCl (*w*/*v*), which has sufficient viscosity for the fabrication of macro-sized beads [24]. Previously, we prepared micro-sized silk sericin particles via an electro-spraying method using this solvent system and investigated the heavy metal removal efficiency of sericin microparticles for wastewater treatment [25,26].

Lignin is one of the main constituents of lignocellulosic biopolymers. It fills the spaces between cell walls of cellulose, hemicellulose and pectin compounds. Nowadays, significant amounts (with a worldwide production of 40–50 million tons per year) of lignin are obtained as a byproduct in the pulping and biofuel production processes [27]. Lignin is an amorphous complex biopolymer that consists of a number of heterogeneous monomers originating from three aromatic alcohols (monolignols): *p*-coumaryl, coniferyl and sinapyl alcohols. The chemical structures of lignin (and thus, the molecular weight, the composition of monomers and thermal properties) vary with the source and isolation process. Because of its heterogeneity, lignin has been used only in low-value applications, such as the generation of heat and electricity. Recently, new methods for the utilization of lignin as a polymeric material have been researched and invented [28,29,30]. Over the past 10 years, many studies have been conducted for the application of lignin for a variety of polymer-based materials, including composites, additives, antioxidants and drug delivery systems [31,32,33,34]. Lignin and lignin-based materials have also been utilized for the removal of organic and inorganic pollutants by many researchers [35,36,37]. Lignin has many functional groups, such as hydroxyls, methoxyl groups, aldehydes, ketones and phenolic groups, which are suitable chelation sites for heavy metal ions. However, most studies on the capability of lignin-based pollutant removal investigated the powder or sieve forms of lignin, which are difficult to use in practical pollutant treatment processes. These forms of lignin require additional processes, including centrifugation, for the separation of the adsorbent from the pollutant. Therefore, the discovery of a recyclable and stable bead-type lignin-based biosorbent will impact both wastewater treatment and the utilization of agricultural by-products.

In the present work, we attempted to prepare a bead-type high-performance Cr(VI)-removal biosorbent. To combine the bead preparation capability of sericin and the high Cr(VI) removal efficiency of lignin, 1 M LiCl in DMSO was used as a solvent system. The effects of blend ratio, pH and metal concentration of the solution, temperature and adsorption time on the adsorption capacity for Cr(VI) were investigated and discussed. Some characterization studies were performed using scanning electron microscopy (SEM), infrared (IR) spectroscopy and energy-dispersive X-ray spectroscopy (EDS) in order to determine the mode of interaction between the Cr(VI) ions and the beads during adsorption. Models fit to equilibrium isotherms and kinetic data are presented here to validate the usefulness of these novel sericin/lignin beads in heavy metal wastewater treatment.

## 2. Results and Discussion

### 2.1. Preparation of Silk Sericin/Kraft Lignin Blend Beads

The bead-forming capability of the kraft lignin (KL) itself was so poor that a stable spherical shape was not maintained through the coagulation process. Therefore, KL was hardly used by itself. To improve the bead-forming capability, silk sericin (SS) was chosen as the base material, because SS not only is easy to use for bead preparation, but also has Cr(VI) adsorption capacity. In this study, we used 1 M LiCl/DMSO as the solvent system, because this solvent system is suitable for dissolution of both protein and lignocellulosic biomaterials.

**Figure 1:**
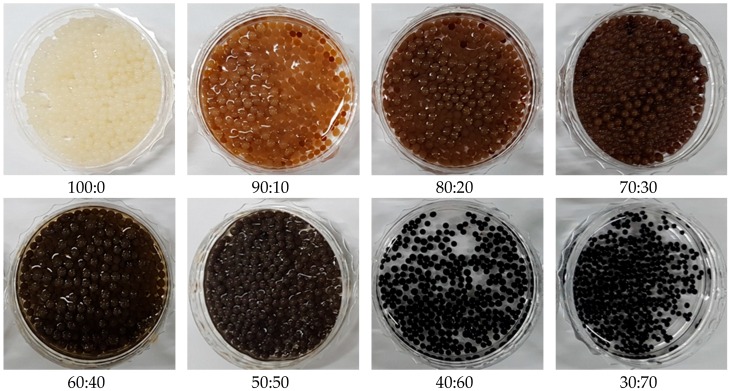
Photos of silk sericin (SS)/kraft lignin (KL) blend beads with various blend ratios.

**Figure 2:**
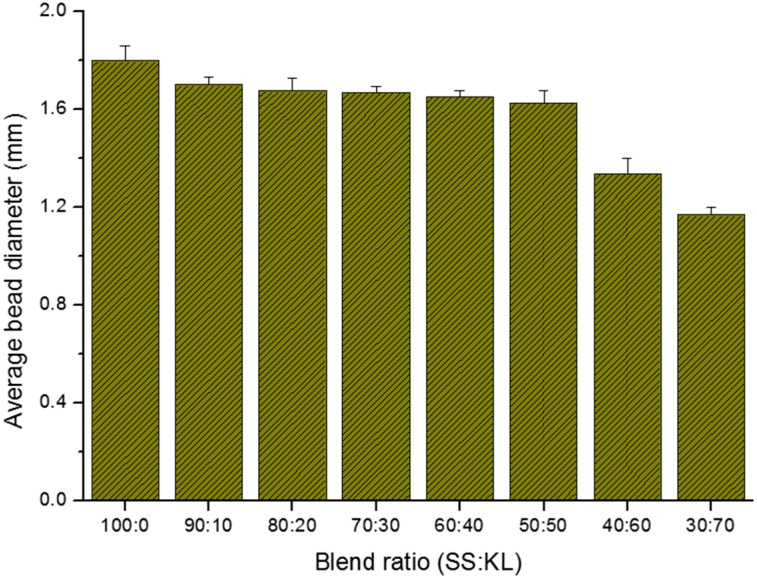
Effect of the SS/KL blend ratio on the average diameter of the as-prepared SS/KL beads.

Before the bead preparation, the only SS solutions were transparent, but darkened when KL content increased. SS/KL beads were prepared based on SS/KL blend ratios ranging from 100:0–30:70. During the coagulation step in methanol, the solution became opaque, and spherical KL/SS beads with a homogeneous surface were fabricated. However, the methanol coagulation bath also became brownish owing to the insufficient coagulation of KL. Figure 1 shows a photo of SS/KL beads with various blend ratios. Spherical beads were prepared successfully with all blend ratios. Figure 2 shows the average diameters of SS/KL beads with various blend ratios. The pure SS beads have an average diameter of approximately 1.80 ± 0.06 mm. As the KL content increased, the average diameter of SS/KL beads decreased. In the case of 50:50 SS/KL beads, the average diameter was 1.62 ± 0.04 mm. However, in the cases of 40:60 and 30:70 beads, the diameters decreased sharply to 1.33 ± 0.06 and 1.16 ± 0.02 mm, respectively. If the KL content increases more than 50% (*w*/*w*), different aspects of the SS/KL bead formation might occur. For a more detailed study, elemental analysis was carried out. SS has a high content of nitrogen because it is a protein, while KL is a carbon-rich molecule with a very small amount of nitrogen atoms. The composition of C, H, N and S atoms in the beads was analyzed, and the result is shown in Table 1. Raw SS beads showed the highest nitrogen contents. The C and S content increased and N content decreased as the KL blend ratio increased up to 50:50. The C/N ratios clearly confirm the incorporation of KL into the resultant beads. The C/N ratios clearly confirm the incorporation of KL into the resultant beads. SS beads showed a C/N ratio of 2.69, and this ratio increased to 5.29 for the 50:50 SS/KL beads. However, the C/N ratios decreased for 40:60 and 30:70 blends, which indicated that there is a blend limitation because of the loss of KL during the coagulation process. Therefore, the decrease of the diameter of SS/KL beads at high KL content might he due to the loss during bead formation.

**Figure 3:**
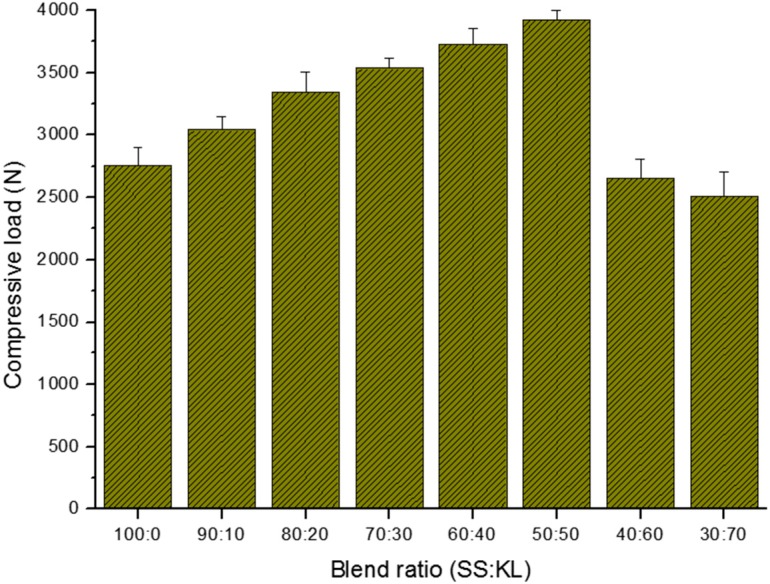
Effect of the SS/KL blend ratio on the compressive load of SS/KL beads.

During the batch-type metal adsorption process, the adsorbent should have suitable mechanical properties to withstand the high-speed agitation or stirring [38,39]. In the case of column-type heavy metal removal processes, the packed biosorbent also tends to be compressed at high flow rates, leading to bead disintegration [40]. It is thus important that the biosorbent possesses sufficient mechanical properties. Lignin has been widely used to reinforce biopolymers in composite materials [41,42]. To investigate the effect of blend ratio on the mechanical properties of SS/KL beads, a compressive strain-stress experiment was carried out, and the average compressive load are shown in Figure 3. The results indicate that the compressive load of SS can be improved by adding KL. The average compressive load of raw SS beads was 2750 N, but the load increased as the concentration of KL increased up to 50% (*w*/*w*) and then decreased dramatically at blend ratios of 40:60 and 30:70. Wang et al. investigated the effect of lignin on the mechanical properties of chitosan fiber and found that an appropriate amount of lignin has a reinforcing effect [43]. This strengthening effect of KL was apparent in this SS/KL bead when the KL content was increased up to 50% (*w*/*w*). Here, the compressive load of SS/KL beads with a high content of KL shows also a significant decrease due to the loss of KL.

### 2.2. Cr(VI) Adsorption onto SS/KL Beads

**Figure 4:**
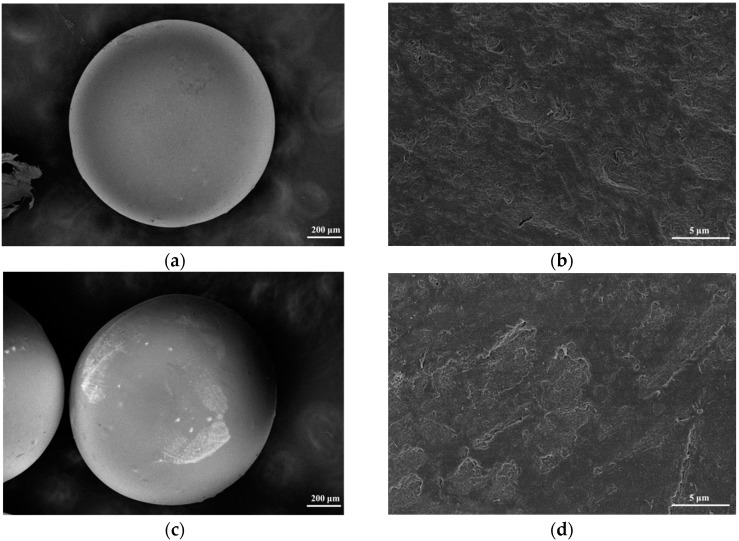
Field emission scanning electron microscopy (FE-SEM) images of SS/KL beads before Cr(VI) adsorption (**a**,**b**) and after Cr(VI) adsorption (**c**,**d**).

**Figure 5:**
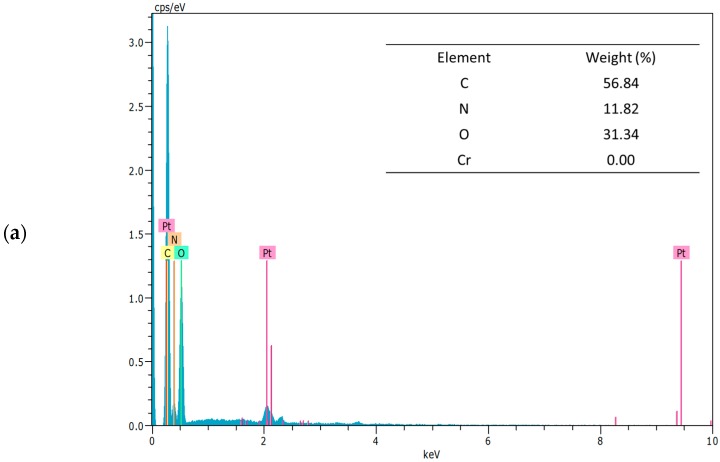

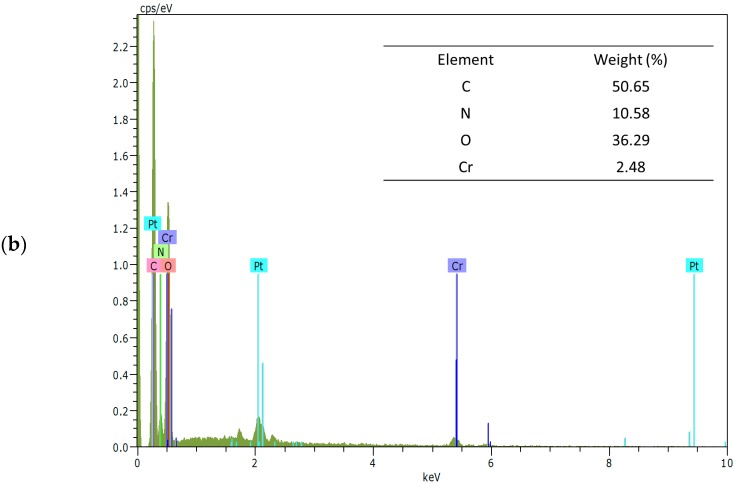
Energy-dispersive X-ray spectroscopy (EDS) spectra and elemental compositions of SS/KL blend beads before Cr(VI) adsorption (**a**) and after Cr(VI) adsorption (**b**).

**Figure 6:**
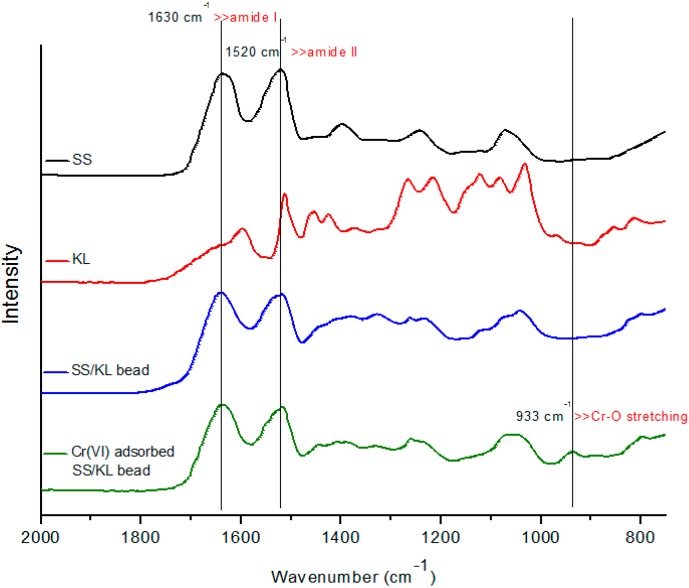
Fourier transform infrared (FTIR) spectra of raw SS, KL powder, SS/KL blend beads and Cr(VI) adsorbed on SS/KL blend beads.

Because Cr(VI) concentration analysis involves a colorimetric method using solutions containing residual Cr(VI) after the adsorption process, it may not represent the real adsorption of Cr(VI) ions onto SS/KL beads. To determine whether the SS/KL beads can adsorb Cr(VI) ions, we examined the SS/KL beads after the Cr(VI) removal process at pH 2 using both EDS and attenuated total reflectance (ATR)-Fourier transform infrared (FTIR) spectroscopy. Figure 4 and Figure 5 show the field emission SEM (FE-SEM) images and EDS spectra before and after the Cr(VI) adsorption experiment. The diameter of the dried SS/KL beads before Cr(VI) adsorption was 0.84 mm, and the beads had smooth surfaces. The C/N ratio of the SS/KL beads from the EDS spectra results was similar to that from elemental analysis. After the Cr(VI) adsorption process, EDS showed that the chromium ion was located on the surface of SS/KL beads, which indicated the capability of SS/KL beads to adsorb chromium. Furthermore, there was no morphological change during the adsorption process, which indicated that the bead maintained structural stability in acidic solution and under mechanical agitation conditions. Figure 6 shows the ATR-FTIR results of SS, KL, SS/KL 50:50 blend beads and SS/KL 50:50 blend beads after Cr(VI) adsorption. The SS powder showed characteristic peptide peaks; both amide I (1700–1600 cm^−1^) corresponding to the stretching vibrations of the C=O bond of amide and amide II (1600–1500 cm^−1^) corresponding to the bending of the N–H bond were observed. In the case of KL, aromatic skeleton vibrations at 1600, 1512 and 1425 cm^−1^ and the C–H deformation combined with aromatic ring vibration at around 1450 cm^−1^ were observed. SS/KL beads shows both of the characteristic SS and KL peaks. There was no change in the amides I and II between SS powder and SS/KL beads, which indicating that KL does not affect the secondary structure of SS. After Cr(VI) adsorption, new peaks at 933 cm^−1^ due to Cr(VI)–O stretching vibration could be found [44]. From the above-mentioned results, it was concluded that the Cr(VI) could be successfully adsorbed on the SS/KL beads.

**Figure 7:**
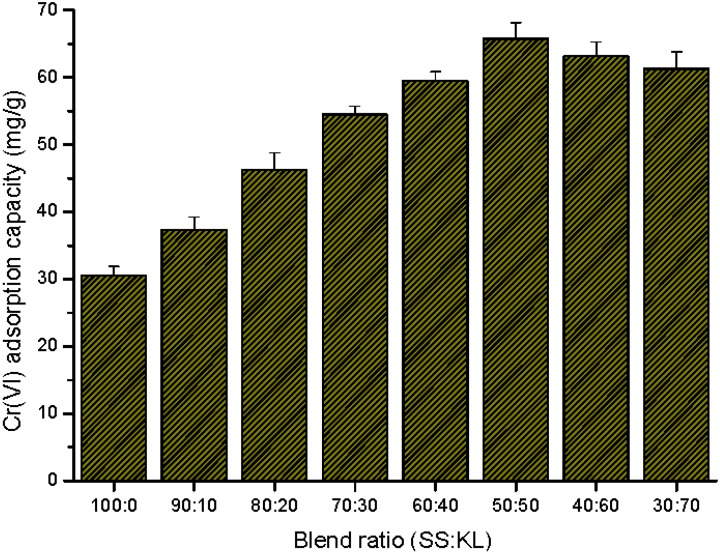
Effect of the SS/KL blend ratio on the Cr(VI) adsorption capacity of SS/KL beads.

The effects of the SS/KL blend ratio on Cr(VI) adsorption capacity are shown in Figure 7. As shown in the data, the SS/KL blend beads showed excellent metal ion adsorption capacity regardless of the blend ratio, compared to SS beads as the control. As the KL content increased from 0% (100:0 SS:KL) to 50% (50:50), the Cr(VI) adsorption capacity increased more than two-fold from 30.48 mg/g for 100:0 beads to 65.72 mg/g for 50:50 beads. This indicated that KL has more functional groups with metal-binding sites than does SS. This increasing effect of Cr(VI) adsorption capacity was weakened in the case of 40:60 and 30:70 blends beads, which have a lower adsorption capacity than 50:50 beads. Based on the physicochemical properties and chromium removal efficiency, 50:50 was the optimal SS/KL blend ratio. For further Cr(VI) adsorption studies, 50:50 blend beads were used.

### 2.3. Cr(VI) Adsorption Behavior

#### 2.3.1. Effect of pH on the Cr(VI) Removal Process

**Figure 8:**
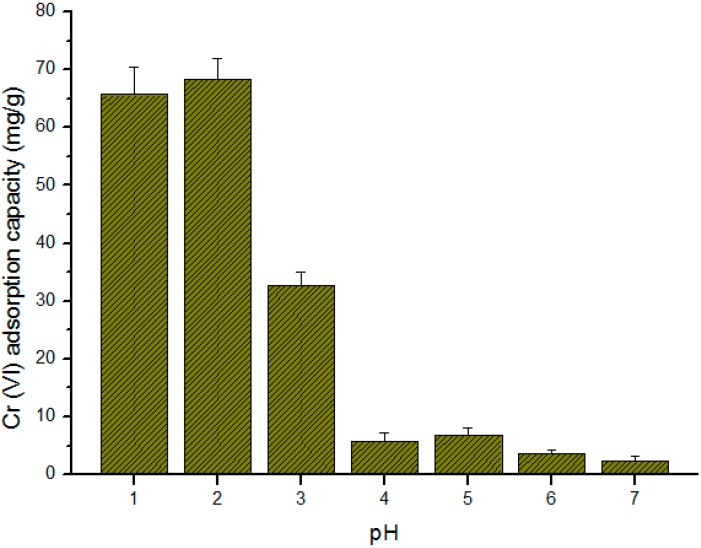
Effect of initial pH on the equilibrium Cr(VI) ion biosorption capacity of SS/KL blend beads (C_0_: 100 mg/L, adsorbent dose: 1.0 g/L, temperature: 25 °C, agitation rate: 180 rpm).

The removal of heavy metal pollutants from wastewater by adsorption and the adsorption efficiency of a biosorbent are highly dependent on the pH of the solution. Generally, the pH of a solution affects not only the surface chemistry of an adsorbent, but also the dissolved metal ion adsorbate species [45]. To determine the effect of pH on the adsorption capacity of SS/KL beads, Cr(VI) adsorption experiments were carried out at the same Cr(VI) concentration (100 mg/L) and SS/KL adsorbent dosage (1 g/L) and different initial pH (from pH 1–7) conditions, and the results are shown in Figure 8. The adsorption capacity of SS/KL beads was higher under strong acidic conditions; the adsorption capacities were 65.98 and 68.42 mg/g at pH 1 and 2, respectively. The values decrease with further increases in solution pH. According to the literature, various Cr(VI) species exist, including HCrO_4_^−^, CrO_4_^2−^ and H_2_CrO_4_, and the distribution is strongly affected by the pH of the solution [46,47]. Under strong acidic conditions, the main species of Cr(VI) is the monovalent anion form, HCrO_4_^−^. The pH also strongly affects the surface charge of SS/KL beads, as well as the protonation degree of the amine group of SS and the phenol group of KL. Figure S1 shows the plot of ΔpH versus initial pH value. The pH point of zero charge (pHpzc) of SS and KL is 4.87 and 5.19, respectively. This means that SS and KL have positive net charges below pHpzc and are negatively charged above this point. Given the overall effect of pH on both the Cr(VI) adsorbate and the bead-type SS/KL adsorbent, under strong acidic conditions, such as pH 1 and 2, the SS/KL beads will have a positive net charge, and there will be strong electrostatic attractions between the negative HCrO_4_^−^ species and the positively-charged SS/KL beads. When pH was increased from 3–7, the monovalent HCrO_4_^−^ species was converted into the divalent CrO_4_^2−^ species, and the positive surface charge of the SS/KL beads also weakened and finally became neutral or negative. Meanwhile, the electrostatic interaction between Cr(VI) species and SS/KL beads decreased; therefore, the adsorption capacity of the beads decreased as the pH of the solution increased from 3–7.

#### 2.3.2. Effect of Initial Concentration and Adsorption Isotherms

**Figure 9:**
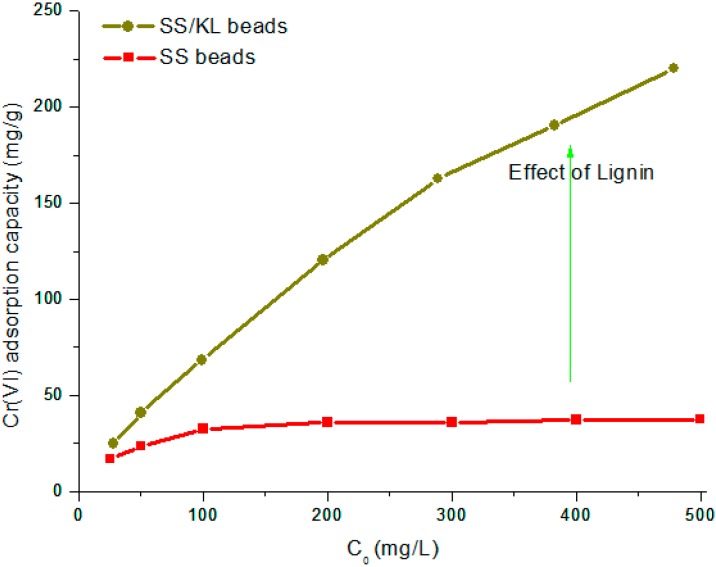
Effect of initial Cr(VI) concentration on the equilibrium Cr(VI) ion biosorption capacities of SS and SS/KL blend beads (adsorbent dose: 1.0 g/L, temperature: 25 °C, agitation rate: 180 rpm).

The effect of initial Cr(VI) concentration on the metal adsorption capacity of the SS/KL beads was investigated by varying the initial Cr(VI) concentration at an optimum pH of 2 and equilibrium time of 24 h. As can be seen in Figure 9, the adsorption capacity of pure SS and SS/KL beads increased with increasing initial Cr(VI) concentration. This phenomenon can be explained through a large driving force for mass transfer. A higher initial Cr(VI) concentration provides a sufficient adsorption environment because it makes the strong driving force to overcome the mass transfer resistance between the Cr(VI) and SS/KL beads. Therefore, a higher initial Cr(VI) concentration could increase the biosorption capacity.

In the case of SS/KL beads, the adsorption capacity did not reach a plateau value as the initial Cr(VI) concentration increased. However, in the case of SS beads, the adsorption capacity reached a plateau, which represents the maximum adsorption capacity of the SS beads. This indicated that the maximum adsorption capacity of SS/KL beads was enhanced owing to the incorporation of the higher adsorption ability of KL in the SS/KL biosorbent. Equilibrium adsorption isotherms show the relationship between the metal concentration in the working solution and the amount adsorbed on the adsorbent. It is important to study the adsorption isotherm from the experimental data to develop an equation that accurately represents the results. It could be helpful to understand the adsorption interface between the adsorbent surface and heavy metal ions. We evaluated the experimental data with the Langmuir, Freundlich and Brunauer–Emmett–Teller (BET) models, which were widely used in analytical isotherm studies. The Langmuir adsorption isotherm assumes that the adsorption process takes place on an energetically-uniform monolayer surface without any interaction between the adsorbed molecules. The Langmuir isotherm is given by Equation (1):

(1) $$q_{e}=\frac{q_{m}K_{L}C_{e}}{1+K_{L}C_{e}}$$

where the notation of *q_e_* is the solid phase equilibrium Cr(VI) concentration (mg/g), *C_e_* is the equilibrium Cr(VI) concentration in the solution (mg/L), *q_m_* is the monolayer biosorption capacity of the adsorbent (mg/g) and *K_L_* is the Langmuir biosorption constant (L/mg), which is related to the free energy of biosorption.

In contrast, the adsorbent that is well matched with the Freundlich isotherm assumes that the adsorption takes place on the heterogeneous adsorbent surfaces. The Freundlich isotherm is expressed as follows:

*q_e_ = K_f_C_e_*^1/*n*^ (2)

where *K_f_* is the Freundlich constant or capacity factor (mg/g) and 1/*n* is the Freundlich exponent; *n* is the heterogeneity factor related to adsorption intensity.

In addition to the Langmuir and Freundlich models, the BET model was used to describe the equilibrium metal biosorption in a batch system. This model assumes the uptake of the metal ions in homogeneous multilayers. This isotherm is expressed using the following equation:

(3) $$\frac{C_{e}}{\left(C_{S}-C_{e}\right)}=(\frac{1}{BQ})+(\frac{B-1}{BQ})(\frac{C_{e}}{C_{S}})$$

where *C_S_* is the saturation concentration of the solute (mg/L), *Q* is the amount of solute adsorbed per unit weight of adsorbent when a monolayer adsorption was completed on the adsorbent surface (mg/L) and *B* is a BET adsorption constant relating to the energy of the surface interaction.

**Figure 10:**
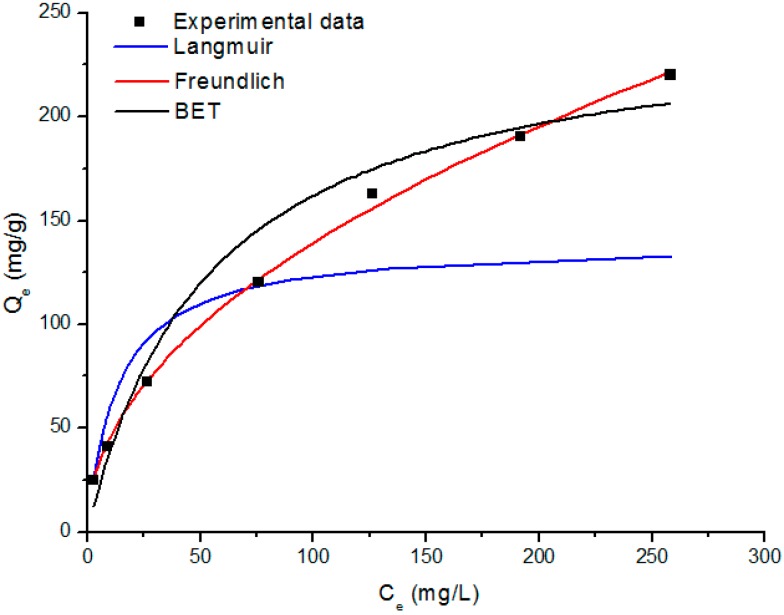
Adjustment of the Langmuir, Freundlich and Brunauer–Emmett–Teller (BET) models to the experimental data obtained from the SS/KL blend beads.

Figure S2 shows the linearized isotherm plots based on the linear forms of each isotherm model equation. Figure 10 shows the Langmuir, Freundlich and BET isotherms obtained by fitting equilibrium data from Figure S2 along with the experimental data. The values obtained for the meaningful parameters and constants of each model are given in Table 2. The regression values (*R*^2^) indicate that the Cr(VI) adsorption behavior of SS/KL beads fits the Langmuir, Freundlich and BET isotherms well. However, from the comparison of the *R*^2^ values, we can conclude that the Freundlich equation represents the best fit for the Cr(VI) adsorption behavior of the SS/KL beads. This result also predicts the heterogeneity of the Cr(VI) adsorption surface of the SS/KL beads. This heterogeneity of the SS/KL biosorbent might be due to the difference in the active adsorption sites between SS and KL. In addition, the Freundlich parameter, *n* value, of SS/KL of 2.05, which is greater than one, indicated that the adsorption behavior is more favorable at a high concentration range, but much less favorable at a lower concentration, which is evidence that the adsorption capacity increases with the increasing initial Cr(VI) concentration [48].

#### 2.3.3. Adsorption Kinetics and Thermodynamics Study

**Figure 11:**
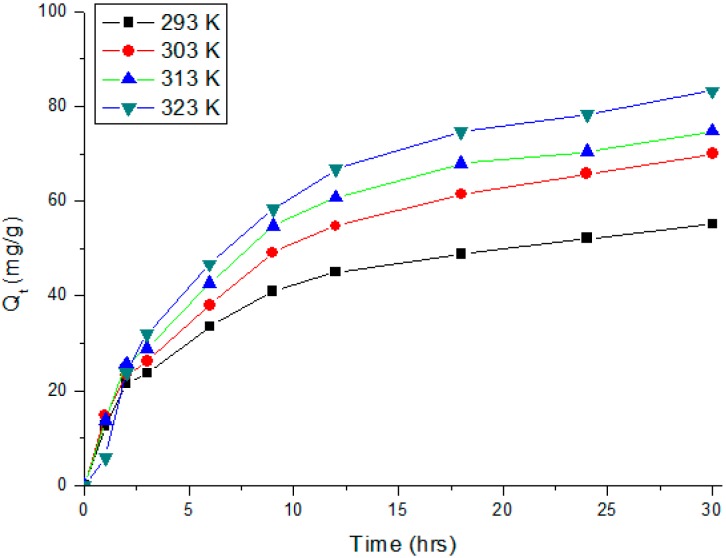
Kinetics curves of Cr(VI) adsorption by SS/KL blend beads at 293, 303, 313 and 323 K.

To study the mechanism of biosorption, the adsorption kinetics was investigated. The experiment involving the adsorption of Cr(VI) on SS/KL beads at pH 2 was carried out, and the adsorption capacity was obtained as a function of time; the results are shown in Figure 11. At the initial adsorption stage, the SS/KL beads showed a fast adsorption rate for Cr(VI) removal. Nearly 50% of the total adsorbed Cr(VI) was adsorbed during the first 5 h. When the contact time was prolonged further, the adsorption rates clearly became slow; finally, equilibrium was achieved at 30 h. In order to find a suitable adsorption kinetics model for the SS/KL beads, the frequently-used models, the pseudo-first-order and pseudo-second-order models, were applied and used to fit the kinetics data. The pseudo-first-order model is expressed as Equation (4):

(4) $$\log(q_{e}-q_{t})=\log q_{e}-\frac{k_{1}}{2.303}t$$

where *q_t_* and *q_e_* are the adsorption capacity of Cr(VI) (mg/g) at time *t* and at equilibrium, respectively. *k*_1_ (min^−1^) is the rate constant of pseudo-first-order adsorption and can be obtained from a plot of $\log\left(q_{e\;}-\;q_{t\;}\right)$ versus (*t*). The pseudo-second-order model is given as Equation (5):

(5) $$\frac{t}{q_{t}}=\frac{1}{k_{2}{q_{e}}^{2}}+\frac{t}{q_{e}}$$

where *k*_2_ is the initial adsorption rate as *t* → 0. According to the above equation, a plot of (*t*/*q_t_*) versus (*t*) will yield a linear plot with a slope of 1/*q*_e_ and an intercept of 1/$k_{2}{q_{e}}^{2}$. The validity of both kinetic models was checked through each linear plot of log(*q_e_* − *q_t_*) against *t* and *t*/*q_t_* against *t*, respectively, and is depicted in Figure S3. The kinetic parameters and regression values for Cr(VI) adsorption are given in Table 3. The higher R^2^ values of the pseudo-second-order model under all temperature conditions suggest that Cr(VI) adsorption on the SS/KL beads is kinetically controlled by a pseudo-second-order rather than a pseudo-first-order kinetics. This indicates that the Cr(VI) adsorption on SS/KL is mainly a chemical adsorption process. This model is usually appropriate for adsorption behavior that occurs through the ion exchange or electrostatic interaction mechanism [49,50,51].

The influence of temperature on the Cr(VI) adsorption behavior of the SS/KL beads is also shown in Figure 11. Experimental results showed that Cr(VI) adsorption capability increased with temperature, similar to the cases of many Cr(VI) adsorption studies. The increase in the adsorption capacity with temperature may be attributed to kinetic effects owing to a larger driving force for the mass transfer and activation of new adsorption sites on the SS/KL bead surfaces at a higher temperature. For more detailed studies, the thermodynamic parameters, such as standard Gibbs free energy (Δ*G*°), standard enthalpy (Δ*H*°) and standard entropy (Δ*S*°) for Cr(VI) adsorption, were estimated. The thermodynamic parameters can be determined from the following equations:

(6) $$\Delta G{}^{\circ}=-\mathrm{RT}\ln K_{C}$$

(7) ΔG°=ΔH°−TΔS°

where the notation of *Kc* is the adsorption equilibrium constant, obtained by multiplying the Langmuir constant *K_L_* and maximum adsorption capacity *q_m_* (mg/g). *R* is the universal gas constant (8.314 × 10^−3^ kJ/(mol·K)) and *T* is the absolute temperature (K). The values of Δ*H*° and Δ*S*° were obtained from the linear plot of ln *Kc* versus 1/*T*, as shown in Figure S4. The calculated thermodynamic parameters are shown in Table 4. The negative values of Gibbs free energy (Δ*G*°) suggest that the Cr(VI) adsorption process is thermodynamically feasible and spontaneous within the temperature range (290–323 K). The Δ*G*° value becomes more negative with increasing temperature, which suggests that higher temperature environments make the adsorption phenomenon easier and more feasible. The negative value of enthalpy (Δ*H*°) indicates the endothermic nature of the biosorption of Cr(VI) onto SS/KL beads. The negative values of entropy (Δ*S*°) represent an increase in randomness at the Cr(VI)-SS/KL bead interface during the adsorption process [52].

#### 2.3.4. Desorption and Regeneration Study

**Figure 12:**
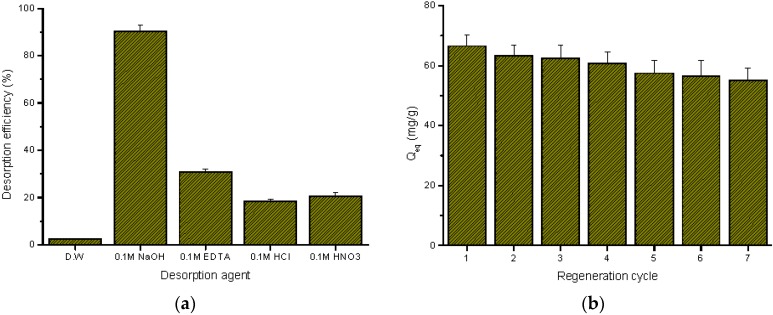
Effect of desorption agent on desorption efficiency (**a**) and its regeneration adsorption capacity of SS/KL blend beads after each cycle (**b**).

From the environmental and economic points of view, the stability and reusability of an adsorbent are very important. Therefore, the most effective desorption condition should be identified. The effect of various desorption agents on desorption efficiency is shown in Figure 12a. The maximum desorption efficiency was observed when 0.1 M NaOH solution was applied. The addition of alkaline solution alters the surface charge of the SS/KL bead from positive to negative (Figure S1), which induces strong repulsive force between negatively-charged SS/KL beads and negative chromate ions. To study the regeneration efficiency of SS/KL beads, the adsorption/desorption process was tested seven times, and its regeneration adsorption capacity is shown in Figure 12b. It can be seen that the Cr(VI) adsorption capacity of SS/KL beads remains at 80% of its initial capacity over seven cycles. This indicates that the SS/KL bead has a good potential for Cr(VI) removal and recovery.

## 3. Materials and Methods

### 3.1. Materials

Silk cocoons were kindly given by National Academy of Agricultural Science (NAAS, Korea). Kraft lignin and analytical reagent grade dimethyl sulfoxide, lithium chloride, methanol, glutaraldehyde solution (25%), potassium dichromate, K_2_Cr_2_O_7_ and 1,5-diphenyl carbazide were purchased from Sigma-Aldrich (Yongin, Korea).

### 3.2. Preparation of Silk Sericin and Kraft Lignin Blend Beads

SS was extracted by boiling 20 g of Bombyx mori silkworm cocoons with 500 mL of distilled water using an autoclave for 1 h at 120 °C. The extracted solution was filtered with a nonwoven filter in order to remove the remaining cocoons. The SS solution was frozen at −70 °C for 4 h and lyophilized. SS/KL beads were prepared using the dripping method, in which the beads were generated by a coagulation process. To prepare the dope solution, the SS and KL solution (22.0%, *w*/*v*) was prepared by dissolving in 1 M LiCl/DMSO solvent. SS solution was mixed with KL solution with various blend ratios of 100:0, 90:10, 80:20, 70:30, 60:40, 50:50, 40:60 and 30:70 by weight. The dope solution was dropped into methanol coagulant through a 26 G syringe using a syringe pump (KD scientific, Holliston, MA, USA). The resultants SS/KL beads were left in the coagulant bath for another 1 h. For the enhancement of water stability and mechanical properties, the crosslinking process was performed with 2% (*v*/*v*) glutaraldehyde (GA) in the same coagulant for 1 h at room temperature. Finally, the SS/KL beads were washed using the same coagulant, followed by washing using distilled water to remove the excess GA.

### 3.3. Characterization of the SS/KL Beads

For the investigated the point of zero charge (P_ZC_), the pH drift method was performed. A total of 50 mL of 0.01 M sodium chloride (NaCl) solution was placed in a closed Erlenmeyer flask. The pH of the solution was adjusted over the range of 2–9 using 0.1 M hydrochloric acid (HCl) or 0.1 M sodium hydroxide (NaOH). Subsequently, 0.1 g of the SS or KL sample were added into the solution. The final pH of the solution was measured after 48 h of agitation and plotted against the initial pH. The pH at the point of intersection of the experimental curve and the line of the initial pH indicates the P_ZC_ of the SS and KL sample. SS/KL beads with various blend ratios were analyzed for their elemental composition in the CHN mode with the elemental analyzer (Flash EA 1112, Thermo Electron Corporation, Waltham, MA, USA). The amount of oxygen was calculated by difference. The compressive load of a single SS/KL beads with various blend ratio beads was measured using a material testing machine (Lloyd Instruments, Ltd., Chichester, UK). After applying 0.05 N to the SS/KL bead, a compression curve was obtained. The compression load was determined from the load at the 50% compressive strain reached. The surfaces of the SS/KL beads were observed using a field-emission scanning electron microscope (FE-SEM), (JSM-7600F, JEOL, Seoul, Korea). Attenuated total reflection Fourier transform infrared spectroscopy (ATR-FTIR, Thermo Scientific, Waltham, MA, USA) was used to identify the Cr(VI) adsorption onto the SS/KL beads. The samples were examined within the wavenumber range of 700–4000 cm^−1^, and 32 scans with 8 cm^−1^ resolution were used to obtain the spectra.

### 3.4. Batch Adsorption Studies

A stock solution of Cr(VI) (1000 mg/L) was prepared in distilled water using an accurate quantity of potassium dichromate, K_2_Cr_2_O_7_ (Sigma-Aldrich Chemical Co., St. Louis, MO, USA). Cr(VI) solutions at other concentrations were prepared from the stock solution by dilution and varied from 25–250 mg/L. To determine the optimum pH for the adsorption process, 0.1 g of biosorbent was added into 100 mL of Cr(VI) solution (100 mg/L). The initial pH values of the Cr(VI) solutions were adjusted from 1.0–7.0 using 1 M H_2_SO_4_ or 1 M NaOH. To compare the adsorption capacity of the raw SS beads and the SS/KL beads, the adsorption experiments were performed under the same conditions. The equilibrium adsorption capacity, *q_e_*, was determined using the following Equation (8):

(8) $$q_{e}=\frac{C_{0}-C_{e}}{M}V$$

where *C*_0_ and *C_e_* are the initial and the equilibrium concentration of the Cr(VI) in the testing solution (mg/L), *V* is the volume of the testing solution (L), and *M* is the weight of the biosorbent (g).

In the biosorption kinetic experiments, 0.1 g of SS/KL beads were added to a 100-mL Cr(VI) solution (100 mg/L). The initial pH was adjusted to 2.0, and the samples were taken at different time intervals.

To obtain the adsorption isotherms, varying initial Cr(VI) concentrations ranging from 25–500 mg/L were used. The batch adsorption equilibrium experiments were conducted in 250-mL Erlenmeyer flasks with 100 mL of the Cr(VI) solution. For all of the adsorption experiments, the flasks were agitated continuously on a multi-stirrer (JEIO Tech, Seoul, Korea) at 180 rpm with the temperature controlled at 25 °C up to 24 h.

### 3.5. Desorption and Regeneration Studies

Various desorption agents, such as distilled water, 0.1 M NaOH, 0.1 M EDTA, 0.1 M HCl and 0.1 M HNO_3_, were used in this study. For the desorption study, after the adsorption experiments, the SS/KL beads were recovered from the Cr(VI) solution using a nonwoven filter. To remove the residual Cr(VI) on the surface, the Cr(VI) adsorbed SS/KL beads were agitated with distilled water on a multi-stirrer for 10 min at 180 rpm, and this washing process was repeated three times. The beads were then soaked in 100 mL of desorption agent, and the mixtures were shaken overnight. The desorption efficiency was calculated as:

(9) $$\mathrm{Desorption}\;\mathrm{efficiency}=\frac{Desorbed\;Cr\;ions\;by\;desorption\;agent}{Adsorbed\;Cr\;ions\;}\times 100$$

To study the recycling efficiency, seven cycles of adsorption-desorption experiments were carried out using 0.1 M NaOH as a desorption agent for 6 h. After each cycle of the experiments, the SS/KL beads were washed three times with distilled water to ensure neutral conditions for the next adsorption-desorption cycle.

## 4. Conclusions

We successfully prepared SS/KL blend beads as a high-performance Cr(VI) bioadsorbent. Owing to the bead formation capability of SS, KL was incorporated directly into the beads during the coagulation process. The Cr(VI) adsorption capacity of SS/KL blend beads increased as the KL content increased. We found that 50:50 (SS:KL) was the optimal blend ratio, which resulted in good mechanical properties and a higher Cr(VI) adsorption capacity.

Agricultural waste is a good candidate material for the removal of heavy metal pollutants because it is inexpensive and easily available in large quantities. SS and KL, the specific waste product of sericulture and the pulping industry meet the demand for new practical applications in the polymeric field. This bead-type SS/KL biosorbent could have potential as a valuable material in the pollutant treatment industry.

## Figures and Tables

**Table 1:** Elemental analysis data of sericin/lignin beads with various blend ratios.

Blend Ratio (Sericin/Lignin)	Elemental Analysis Data Calculated Value (%)			
	C	H	N	S
100:0	39.39	6.42	14.64	0.40
90:10	40.84	6.28	12.53	0.52
80:20	41.35	6.42	12.06	0.64
70:30	42.51	6.53	11.33	0.80
60:40	44.40	6.52	10.67	0.92
50:50	46.78	6.50	8.96	1.04
40:60	45.21	6.30	9.76	1.01
30:70	45.33	6.44	9.56	0.94
Kraft lignin powder	61.60	6.27	0.50	1.75

**Table 2:** Isotherm constants and correlation coefficients for the biosorption of Cr(VI).

Parameter	Value	*R*^2^
**Langmuir isotherm**	–	0.916
*Q* (mg/g)	139.86	
*K_L_* (L/mg)	0.074	
**Freundlich isotherm**	–	0.999
*n* (L/mg)	2.05	
*K_f_* (mg/g)	14.72	
**BET isotherm**	–	0.952
*Q* (mg/g)	250.10	
*B* (g/mg)	2.26 × 10^5^	

**Table 3:** Kinetic parameters of the pseudo-first-order and pseudo-second-order models for Cr(VI) adsorption onto SS/KL beads.

C_0_ (mg/L)	Pseudo-First-Order			Pseudo-Second-Order		
100	***K*_1_** **(min^−1^)**	***q_e_*** **(mg·g^−1^)**	***R*^2^**	***K*_2_** **× 10^−3^ (g·mg^−1^·min^−1^)**	***q_e_*** **(mg·g^−1^)**	***R*^2^**
	2.65 × 10^3^	53.62	0.791	0.0137	53.16	0.998

**Table 4:** Isotherm constants and correlation coefficients for the biosorption of Cr(VI).

Temperature (K)	Δ*G*° (kJ/mol)	Δ*H*° (kJ/mol)	Δ*S*° (kJ/mol·K)
293	−0.511	10.53	0.121
303	−2.133		
313	−2.827		
323	−4.300		

## Supplementary Materials

Supplementary materials can be found at www.mdpi.com/1422-0067/17/9/1466/s1.
