# Supplementary material for: Preparation of Silk Sericin/Lignin Blend Beads for the Removal of Hexavalent Chromium Ions
Source: Int J Mol Sci. 2016 Sep 2;17(9):1466. doi: 10.3390/ijms17091466 (PMC5037744; doi:10.3390/ijms17091466)
Supplement: Supplementary file 1 [file ijms-17-01466-s001.pdf]

Supplementary Materials: Preparation of Silk Sericin/Lignin Blend Beads for the Removal of Hexavalent Chromium Ions

Hyo Won Kwak, Munju Shin, Haesung Yun and Ki Hoon Lee

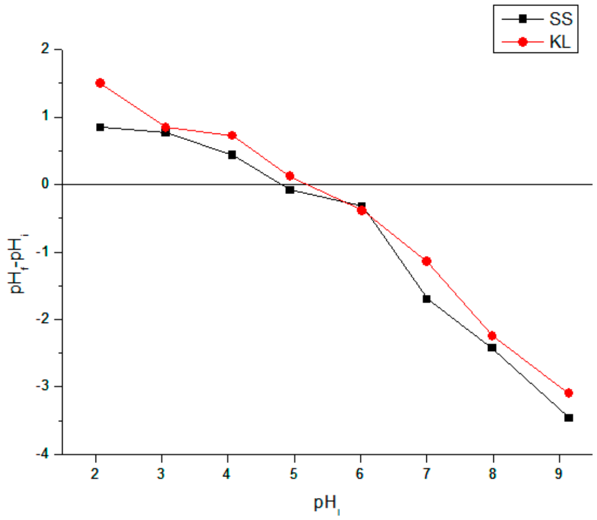

Figure S1. Point of zero charge (pH_{pzc}) of the SS and KL, determined by the pH drift method.

(a)

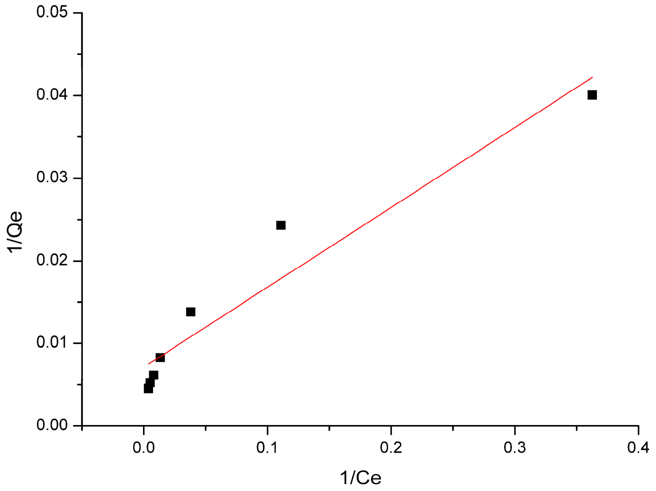

(b)

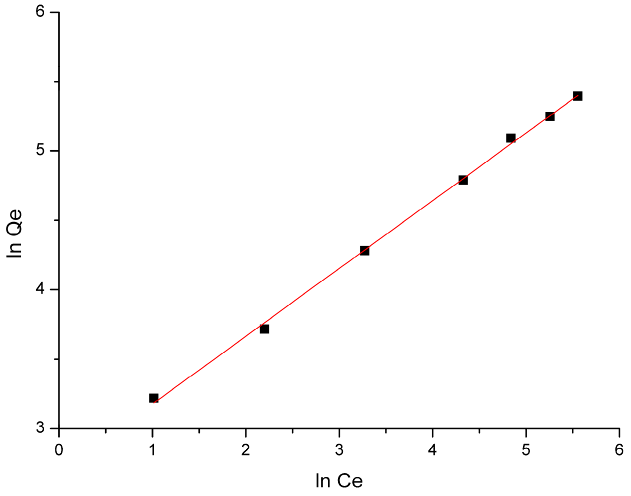

Figure S2. *Cont.*

(**c**)

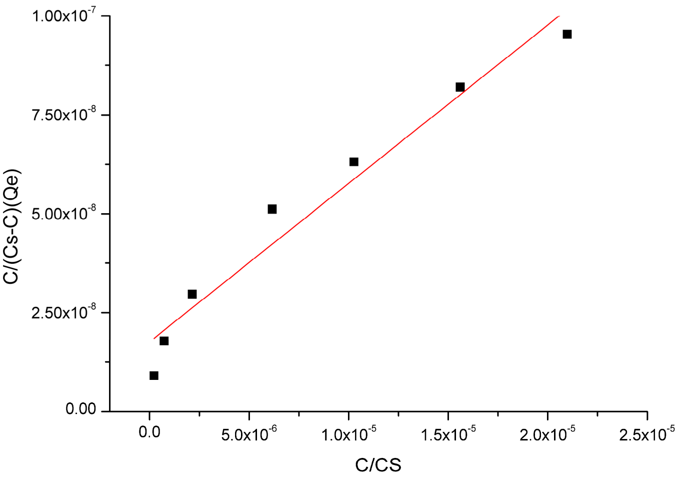

Figure S2. Linear plot of (**a**) Langmuir, (**b**) Freundlich and (**c**) BET isotherms of SS/KL beads.

(**a**)

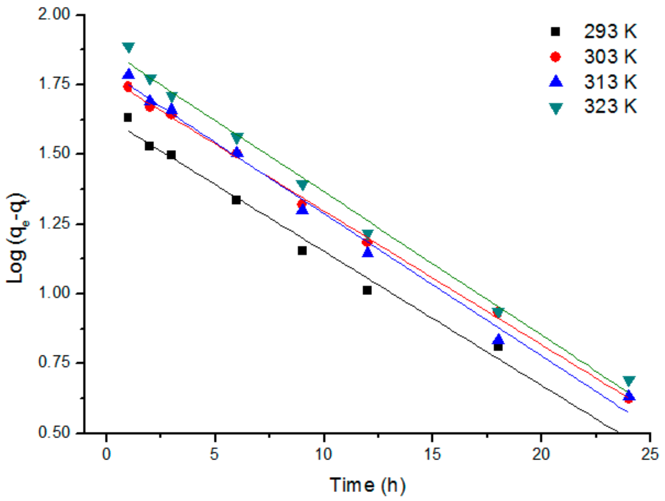

(**b**)

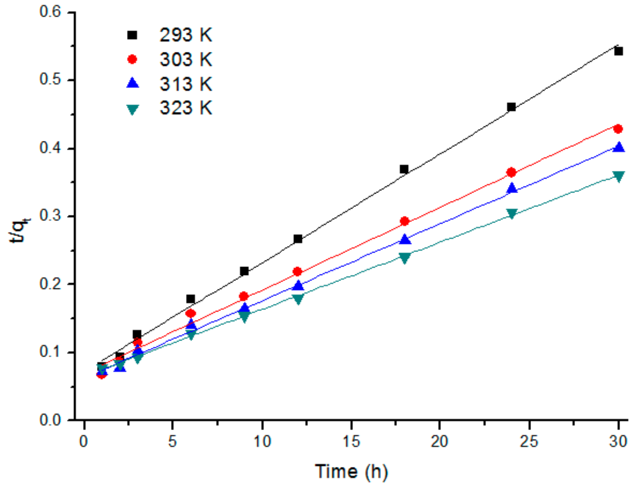

Figure S3. Linear plot of (**a**) the pseudo-first-order and (**b**) the pseudo-second-order kinetics of SS/KL beads.

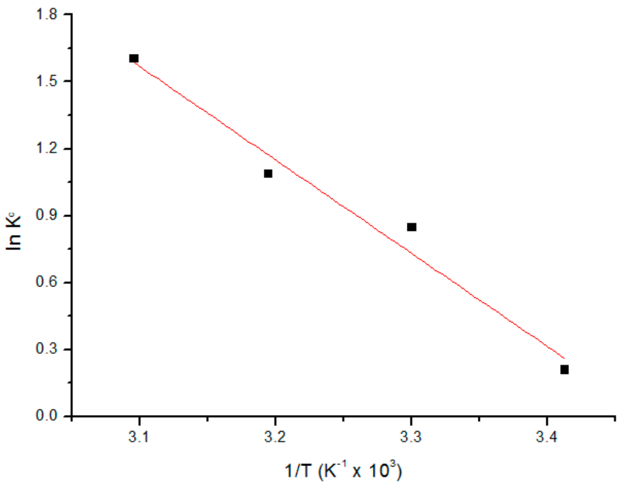

Figure S4. Plot of ln Kc versus 1/T for the estimation of the thermodynamics of SS/KL beads.
